# Precision Comparison of the Lattice Parameters of Silicon Monocrystals

**DOI:** 10.6028/jres.099.002

**Published:** 1994

**Authors:** E. G. Kessler, A. Henins, R. D. Deslattes, L. Nielsen, M. Arif

**Affiliations:** National Institute of Standards and Technology, Gaithersburg, MD 20899-0001

**Keywords:** Bragg angle, lattice spacing, silicon, x-ray diffraction, x-ray spectrometer

## Abstract

The lattice spacing comparator established at the National Institute of Standards and Technology to measure the lattice spacing differences between nearly perfect crystals is described in detail. Lattice spacing differences are inferred from the measured differences in Bragg angles for different crystals. The comparator is a two crystal spectrometer used in the nearly nondispersive geometry. It has two x-ray sources, two detectors, and a device which permits remote interchange of the second crystal sample. A sensitive heterodyne interferometer which is calibrated with an optical polygon is used to measure the Bragg angles. The crystals are manufactured with nearly equal thicknesses so that the recorded profiles exhibit pendellosung oscillations which permit more precise division of the x-ray profiles. The difference in lattice spacing between silicon samples used at Physikalisch-Technische Bundesanstalt (PTB) and NIST has been measured with a relative uncertainly of 1 × 10^−8^, This measurement is consistent with absolute lattice spacing measurements made at PTB and NIST. Components of uncertainty associated with systematic effects due to misalignments are derived and estimated.

## 1. Introduction

Absolute measurements of the crystal lattice spacing of silicon have beert reported by two laboratories [1,2,3,4] and are in progress in at least two other laboratories [5,6]. These experiments present a very difficult metrological challenge and require extreme care in order to achieve a relative uncertainty of several parts in 10^8^. The output of these experiments is the lattice spacing of a particular silicon specimen in terms of an optical wavelength standard.

Crystals having well defined lattice spacings and whose lattice spacings are known with an uncertainty in the 0.1 to 0.01 ppm range (1ppm = 10^−6^) are needed in the field of metrology (i.e., x-ray and gamma-ray wavelength measurements). However, the effort required to directly compare a large family of crystal samples to an optical wavelength standard is prohibitive. Thus, several techniques and devices to more rapidly measure lattice spacing differences with a relative uncertainty below 0.1 ppm have been devised. This paper describes in detail the precision lattice spacing comparator (Δ*d* comparator) established at the National Institute of Standards and Technology and reports the results of a careful comparison of Si samples which were measured absolutely by the x-ray interferometer technique at the Physikalisch-Technische Bundesanstalt (PTB) and NIST. The measured lattice spacing difference has a relative uncertainty of 1.4 × 10^−8^ and is, at present, consistent with the absolute measurements reported by the two laboratories.

## 2. Principle of the Measurement

**Fig. 1:**
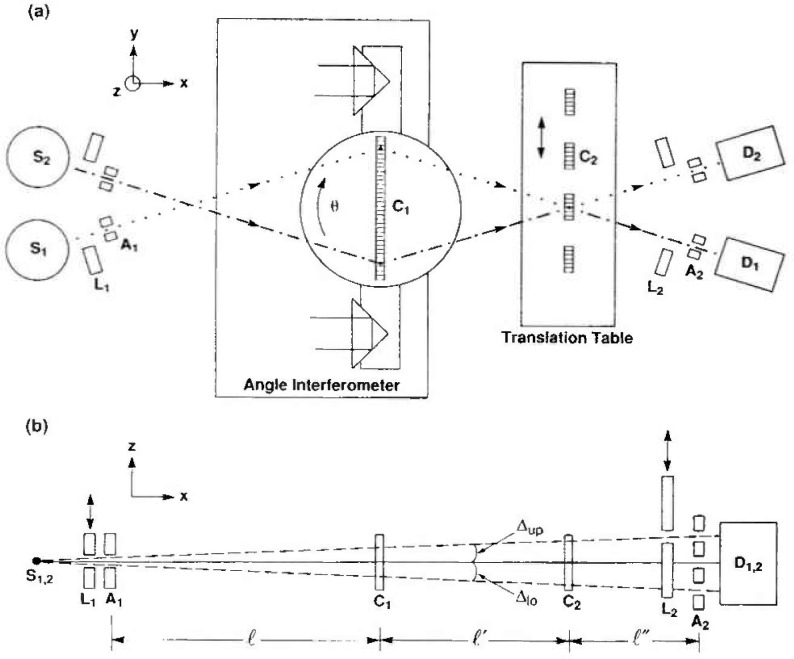
Schematic diagram of the NIST lattice spacing comparator. S_1_, S_2_–x-ray sources; L_1_, L_2_–shutters; A_1_, A_2_–apertures; C_1_, C_2_–crystals; D_1_, D_2_–detectors; *l*, *l*′ *l*″–*x*-axis projections of the x-ray paths from A_1_ to C_1_, C_1_ to C_2_, C_2_ to A_2_, respectively. a) Top view (*x*, *y* plane), b) side view (*x*, *z* plane)

The principle of the NIST lattice-comparison measurement is illustrated by the geometrical layout of the spectrometer (Fig. 1). This geometry, which is similar to one suggested by Hart [7], is a conventional double-crystal Laue-case spectrometer. Ando et al. [8], Becker et al. [9], and Häusermann et al. [10] have also used similar geometries to precisely compare lattice spacings. As will emerge in the discussion which follows, the NIST instrument has certain new features which enhance its generality and sensitivity. The spectrometer has two sources, S_1_ and S_2_, two crystals, C_1_ and C_2_ and two detectors, D_1_ and D_2_, and is used to record only nearly nondispersive profiles. The x-ray beams are defined by precision source, A_1_, and detector, A_2_, apertures and there are computer controlled shutters, L_1_ and L_2_, near the sources and the detectors, respectively.

By using the spectrometer in the nearly nondispersive mode, the large intrinsic linewidths associated with characteristic x-ray emission do not contribute appreciably to the width of the recorded profiles. The source shutters permit isolation of the two x-ray paths so that doubly diffracted profiles from one source can be recorded without interference from singly diffracted profiles from the other source. There are three apertures in front of each detector (Fig. 1b), one above, one on, and one below the plane of dispersion. An indexed shutter selects one of these apertures depending on whether lattice spacing difference scans or crystal alignment scans are being recorded. For lattice spacing difference scans, apertures on the plane of dispersion are used for both sources and the detectors. As crystal 1 is rotated through the reflection, the source shutters alternately pass and block the two x-ray paths, path (⋯) and path (–•–), so that the two x-ray profiles are essentially recorded simultaneously. Thus errors due to drifts of the crystals or the angle interferometer are eliminated. For crystal alignment scans, only one source is used and the detector shutter alternately permits radiation to pass through the apertures above and below the plane of dispersion as crystal 1 is scanned. The angular shift of these two out-of-plane profiles is related to the tilt misalignment of the planes of the first crystal with respect to the planes of the second crystal. By careful alignment, the contribution to the uncertainty due to crystal tilt misalignment can be made small (see Appendix A).

General features of the comparator operation may be understood as follows: If the crystal planes near the ends of the first crystal are parallel and the lattice spacings of the first (*d*_1_) and the second (*d*_2_) crystals are equal (*d*_1_ = *d*_2_), then the profiles in the path (⋯) and the path (–•–) will peak at the same angular setting of the first crystal. However, if *d*_1_*≠d*_2_, then there will be an angular offset, *β*_2,1_ between the two profiles which is a measure of the lattice spacing difference. Δ*d = d*_2_−*d*_1_, between the two crystals. If *θ*_1_ and *θ*_2_ are the Bragg angles of crystals 1 and 2, respectively, then *β*_2,1_ *= 2*(*θ*_2_−*θ*_1_) = 2Δ*θ*. If Δ*d/d* is small (<10^−5^) and the required uncertainty in Δ*d/d* is ~10^−8^, then it is appropriate to use the differential form of the Bragg equation to express Δ*d/d.*

$$\Delta d/d=-(\Delta \theta )\;\cot\;\theta =-(\beta_{2,1}/2)\cot\theta$$ (1)

In our comparison scheme, the first long crystal serves as a temporary (a few hours) reference. Profiles are recorded using one of the small second crystals. Then another small second crystal is brought into the position where the x-ray beams diffracted by the first crystal intersect. The first crystal is again scanned and profiles are recorded.

By subtracting the angular offsets, *β*, measured with the different second crystal samples, the properties of the first crystal such as the absolute lattice spacing, the effects of variation in lattice spacing, and parallelism of crystal planes at the two ends of the crystal are eliminated. One needs only assume that the properties of the first crystal are constant during the time of measurement of the two different second crystal samples. The equation for the difference in crystal lattice spacings for the pair of small crystals becomes

$$\Delta d/d_{s}=-(\Delta \theta )\;\cot\;\theta_{s}=-(\beta /2)\cot\theta_{s},$$ (2)

where *θ*_s_ = the Bragg angle for the standard sample, *θ*_u_ = the Bragg angle for the unknown sample, *d*_s_ = lattice spacing of standard sample, *d*_u_ = lattice spacing of unknown sample, Δ*d = d*_s_*−d*_u_, *β*_s_ = 2(*θ*_s_*−θ*_l_) = angular offset for the standard sample, *β*_u_ = 2(*θ*_u_−*θ*_l_) = angular offset for the unknown sample, and *β = β*_s_*−β*_u_ = 2(*θ*_S_*−θ*_u_) = 2Δ *θ*.

In Appendix A the equations for calculating Δ*d* in ideal and misaligned geometries are derived. For small lattice parameter differences and well aligned x-ray beams and crystals, the more involved equations derived in the appendix reduce to Eq. (2). The forms of the corrections associated with misalignments are explicitly given and their magnitudes are estimated for our particular spectrometer. This appendix is likely to be most interesting to the precision x-ray specialist. Throughout this paper and particularly in the appendix the path (⋯) is referred to as the path (−) and the path (–•–) is referred to as the path (+).

Since x rays from the two paths interrogate the same area of the second crystal, small variations in lattice spacing along a crystal can be measured with this scheme. The second crystal samples reside on a precision slide which allows easy interchange of crystal samples. A typical data sequence involves many changes of crystal samples and permits compensation for drifts of the crystals and the angle measuring spectrometer.

## 3. Experimental Apparatus

The lattice comparison spectrometer rests on a 1 m × 2 m cast iron plate which is isolated from building vibrations by three passive air bags. The x-ray tubes, detectors, and shutters are rigidly attached to this plate. A smaller plate (61 cm × 122 cm) on which the laser, receiver, and precision spectrometer are mounted rests on vibration damping rubber feet on top of the large cast iron plate. The precision spectrometer is constructed on a 35 cm × 74 cm × 25 cm thick cast iron U channel. The angle interferometer, rotary table, and translation table reside on top of the inverted U channel and the drive arm coupled to the rotary table is conveniently located underneath the channel. The important dimensions which define the scale of the spectrometer are: 1) x-ray focal spot to source slit = 6 cm; 2) source slit to x-ray tube pivot = 25.4 cm; 3) x-ray tube pivot to first crystal = 8.25 cm; 4) first crystal to second crystal = 8.25 cm; and 5) second crystal to detector slit = 28.5 cm.

The radiation sources are identical silver or molybdenum x-ray tubes operated at *V*=40 keV and *I* = 15 mA and having a 1 mm × 1 mm focal spot (in projection). The tubes are cooled by temperature regulated water so that the spectrometer temperature remains constant and uniform. The x-ray sources pivot about the point of intersection of the x-ray beams which lies between the sources and the first crystal. Bragg angles from 10° to 22° are accessible. The vertically defining slits in front of the sources have a 1 mm opening. The center of these slits and the middle slits near the detectors are carefully placed on the plane of dispersion (plane perpendicular to the axis of rotation) to within 0.1 mm. The x-ray tubes are positioned so that the focal spots fill the source and detector slits uniformly. The lead shutters near the source slits are computer controlled to block or pass the x-ray beams.

The axis of rotation is a precision commercial rotary table [11] having a radial concentricity and axial movement less than one micrometer. The table is driven by a stepping motor coupled to a threadless screw and a 48 cm long tangent arm with fine adjustment of the rotation obtained by piezoelectric transducers. The long first crystal is mounted in the center of the rotating table so that its diffracting planes are parallel to the axis of rotation to within a few seconds (of plane angle).

**Fig. 2:**
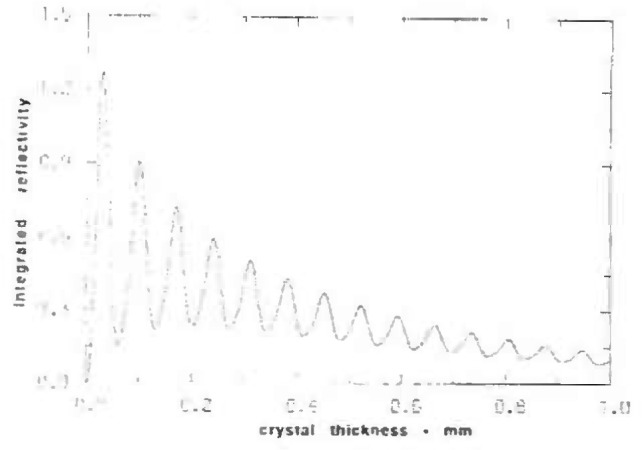
Schematic diagram of the angle interferometer. a, c, g, h – beam steering elements: b–polarization sensitive beam splitter; c. d – 90° polarization rotators; f – glass path compensating plate: i. j – roof prisms; k, l – corner cubes. The insert shows the passage of the beam through the corner cube – roof prism part of the interferometer.

Angles are measured by a polarization encoded Michelson interferometer having an angular sensitivity of a few × 10^−4^ seconds. The interferometer is shown schematically in Fig. 2 where the inset shows the over and under arrangement of the beams. The interferometer is illuminated by a commercial 633 nm HeNe laser [12] which emits two orthogonal linearly polarized frequencies separated by 1.8 MHz. The polarization sensitive beam splitter, b, divides the incoming light beam so that one polarization and frequency traverses one arm of the interferometer and the other polarization and frequency traverses the other arm. The beam splitter, steering elements (e, g, h), and compensating element (f) are chosen and positioned so that the two interferometer arms have equal air and equal glass paths at zero angle. Elements c and d are 90° polarization rotators which rotate the plane of polarization of the outgoing and return beams respectively in order that both corner cubes are transversed with the same polarization orientation and that the output beams are directed away from the laser. The roof prisms, i and j, return the light to the corner cubes, k and l, doubling the angular sensitivity of the interferometer and keeping the output beams fixed in space. The corner cubes are mounted on an arm which is rigidly attached to the rotating crystal table and retroreflect the light in each arm of the interferometer.

The interferometer output beam which includes the two orthogonally polarized frequencies is analyzed by a 45° polarizer included in a commercial detector which provides a measurement difference frequency signal. The laser and its electronics also provides a reference difference frequency signal for the light emitted by the laser. For a stationary interferometer the two difference frequency signals are identical except for a phase difference ϕ, which is proportional to the difference in the optical path lengths of the two interferometer arms. Rotation of the interferometer arm causes a phase shift of the measurement difference frequency signal relative to the reference difference frequency signal. Conversely, phase comparison of the measurement difference frequency signal with the reference difference frequency signal permits measurement of the angular rotation.

The 1.8 MHz difference frequency signals must be converted into signals which permit up-down counting for clockwise and counter clockwise rotation of the axis, measurement of fringe fractions, and servo control of the axis position. Although electronics to perform these functions is commercially available, no existing system could be conveniently coupled to our spectrometer. An electronics module to perform the above functions has been developed and is briefly described in Appendix B.

The diffraction profiles are recorded as x-ray intensity vs interferometer fringes and, thus, the angular offsets, *β* are also measured in interferometer fringes. However, the angular offsets, *β*, needed in Eq. (2) must be in absolute angles. In order to convert interferometer fringes into absolute angles, the angle interferometer is calibrated using an optical polygon and a photoelectric autocollimator [4]. A 24-sided optical polygon (external angles ~15°) is mounted on top of an indexing table on the rotation axis in place of the first crystal. The photoelectric autocollimator senses the directions normal to the faces of the optical polygon. The 24 external angles are measured in terms of interferometer fringes and the sum of the angles is constrained to be 360°. The equation relating angles and fringes is *F=K* sin(*θ*) where *F* is the fringe number, *θ* is the corresponding angle of rotation, and *K* is the calibration constant. The measured value of *K* is 5138551.7 at 22.5 °C. Near *θ* = 0°, the value for the angular rotation/fringe is 0.0401 arcsec/fringe. The calibration constant is directly related to the separation of the corner cubes. Because the arm which defines the separation of the corner cubes is made out of stainless steel, the calibration constant has a large temperature coefficient (~ 16.7 ppm/°C). As long as the angular offsets are small and the temperature is well controlled, this large temperature coefficient can he tolerated. However, in the near future the spectrometer will be equipped with an invar corner cube arm.

The zero angle of the interferometer is determined by measuring one of the external angles of the polygon in a symmetric and asymmetric fashion. In the symmetric measurement, the polygon is positioned on the axis so that the faces forming the 15° angle are measured at +7.5° and −7.5°. This symmetric measurement of the external angle is quite insensitive to the zero angle of the interferometer. In the asymmetric measurement, the polygon is positioned so that the faces of the same angle are measured at ±14.5° and ∓0.5°. This asymmetric measurement of the external angle is very sensitive to the zero angle of the interferometer. The zero angle is determined by requiring equality of the two measurements of the same angle. The uncertainty of the zero angle determination is approximately 2 arcsec which makes a completely negligible contribution to the measurement of the small angular offsets near zero angle.

The second crystals reside on a precision translator equipped with a stepping motor and a linear encoder [13]. The slide has 15 cm of travel and a positioning accuracy of 0.01 mm. Although the slide has pitch and yaw of a few seconds in a few cm of travel, it is reproducible at the 0.01 second level and shows no short term drift. Each second crystal is mounted on a flexure hinge so its planes can be oriented to be roughly parallel to the planes of the first crystal. Fine adjustment is achieved by applying voltage to a piezoelectric “tipper.”

The diffracted x-rays are detected by two identical NaI(Tl) detectors which pivot about the second crystal position. In front of each detector are three slits each 1.6 mm × 1.6 mm. The slits are arranged in the direction normal to the plane of dispersion with center of the middle opening on the plane of dispersion and the top and bottom openings 3.2 mm above and below the plane of dispersion. Each slit has a computer controlled shutter which permits radiation to pass through any one of the three openings.

## 4. Crystal Preparation

In order to make measurements with an uncertainty of 0.01 ppm, considerable care must be exercised in cutting, polishing, mounting, and aligning the crystal samples. The full width at half maximum (FWHM) of nondispersive x-ray profiles obtained with Ag Kα radiation and the Si 440 reflection is about 0.6 seconds. Since the angular separation which corresponds to a lattice spacing difference of 1 × 10^−8^ is ~0.001 seconds, it appears that peak positions must be determined to 1/500 of the FWHM. This is a formidable task requiring very reproducible profiles. However, by carefully matching the thickness of the first and second crystals to within a few micrometers, nondispersive Laue-case rocking curves exhibit oscillatory fine structure which has a modulation period which is typically less than 1/10 of the FWHM of the profile. This fine structure is predicted by the dynamical theory of x-ray diffraction and has been used in high precision determinations of the structure factor of Si [14, 15] and Ge [16] and in x-ray refractive index measurents [17]. We use this fine structure on the x-ray profiles as a sharp convenient reference to measure the angular separation of the profiles.

**Fig. 3:**
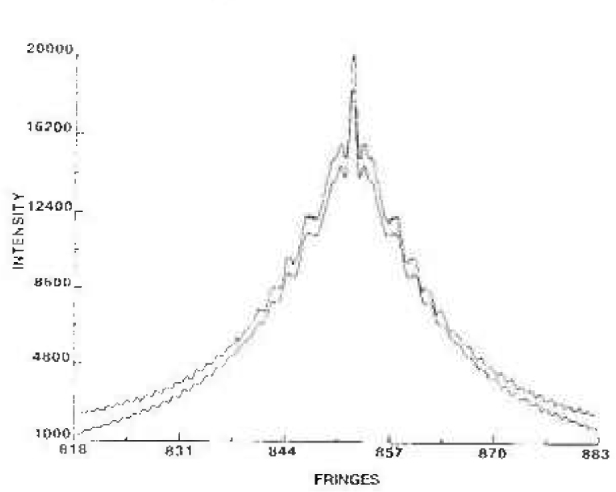
Integrated reflectivity of double crystal nondispersive profiles as a function of crystal thickness for the Si 440 reflection and Ag Kα_1_ radiation.

**Fig. 4:**
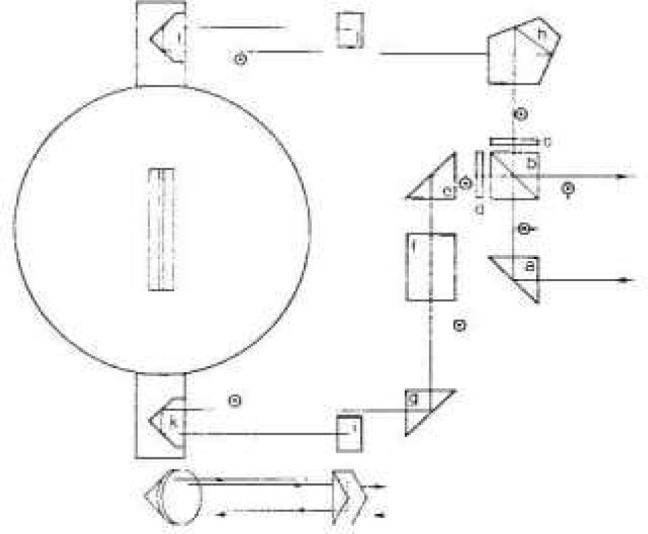
Theoretical (lower) and experimental (upper) profiles for the 440 reflection of Ag Kα radiation using 0.455 mm thick Si crystals.

The integrated reflectivity for the double crystal profiles oscillates as a function of crystal thickness. In Fig. 3, the integrated reflectivity vs crystal thickness is shown for the Si 440 diffraction of Ag Kα radiation. We have chosen a crystal thickness of 0.455 mm as a compromise of intensity and practical problems with thin crystals. Theoretical and experimental profiles for this thickness are shown in Fig. 4.

The theoretical profile [18, 19] is obtained by adding the convolution of two intrinsic reflections for the two polarization states (*σ* and *π*)

$$I(\Delta )=I_{0}\sum_{\sigma ,\pi }\int I^{II,\sigma }(\theta )I^{\pi ,\sigma }(\theta +\Delta )d\theta$$

where

*Δ* = the angle between the diffracting planes of crystal 1 and crystal 2
*I^π,σ^*(*θ*) = exp(− *μt*/cos*θ*_B_)|sin[*A*(*y*^2^+*v*^2^)^1/2^]/(*y*^2^+*v*^2^)^1/2^|^2^
*A =πt/t*_0_
*x*_h_ = *x*_hr_+*ix*_hi_
*y =Δ* sin2*θ*_B_/(*C*|*x*_hr_|)
$v^{2}=x_{h}x_{\bar{h}}/|x_{h}x_{\bar{h}}|$
*t*_0_ = *λ* cos *θ*_B_/(*C*|*x*_hr_|) $C=\{\begin{array}{cc} 1 & \sigma \;\text{polarization}\\ \left|\cos2\theta_{B}\right| & \pi \;\text{polarization} \end{array}$

and

*θ*_B_ is the Bragg angle
*μ* is the linear absorption coefficient
*t* is the crystal thickness
*t*_0_ is the extinction length
*y* is the angular parameter
*C* is the polarization factor
*λ* is the wavelength
*x*_h_ is the complex electronic susceptibility of the crystal: *x*_hr_*+ix*_hi_

For low absorption where *x*_hr_ *> x*_hi_, it can be shown that

$$\begin{array}{c} v^{2}=1-i(\epsilon \mu t_{0})/(\pi \cos\theta_{B})\\ t_{0}=\pi V\cos\theta_{B}/(\lambda r_{c}F|C|)\\ I^{\pi ,\sigma }(\theta )=[\exp(-\mu t/\cos\theta_{B})/(y^{2}+1)]\\ \left\{[\sin^{2}[A(y^{2}+1{)}^{1/2}]+\sin h^{2}[\mu t\epsilon (y^{2}+1{)}^{-1/2}/2\cos\theta_{B}]\right\} \end{array}$$

where

*ϵ* (|*x*_hi_|/|*x*_oi_|)|*C*|
*x*_hi_, *x*_oi_ = imaginary parts of the Fourier coefficients *x*_h_, *x*_o_
*V*= volume of the unit cell
*r_c_* = classical electron radius
*F*= temperature modified structure factor

When generating a theoretical profile, numerical values must be assigned to the following quantities *μ, t, F*, λ, *r_c_, V*, and *ϵ*. Conversely, when fitting an experimental profile with the theoretical description, a quantity such as *t* or *F* can be varied until the difference between the experimental and theoretical profiles is a minimum.

**Fig. 5:**
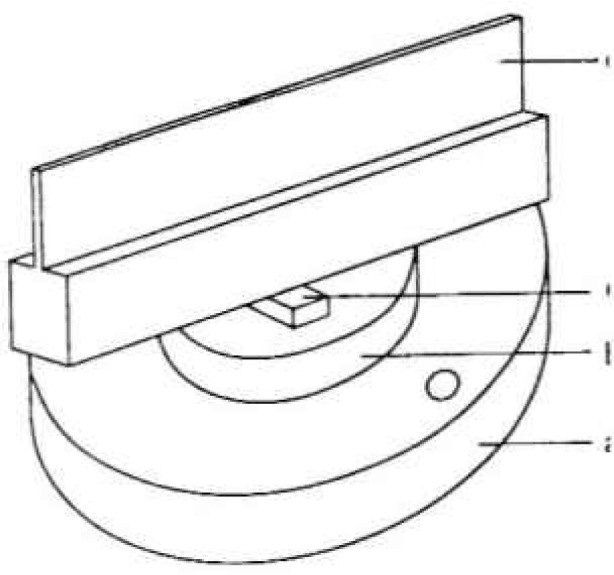
Detailed drawings of the first crystal, a – cast iron, b – Si disk, and c – Si rod, and d-Si diffraction crystal.

**Fig. 6:**
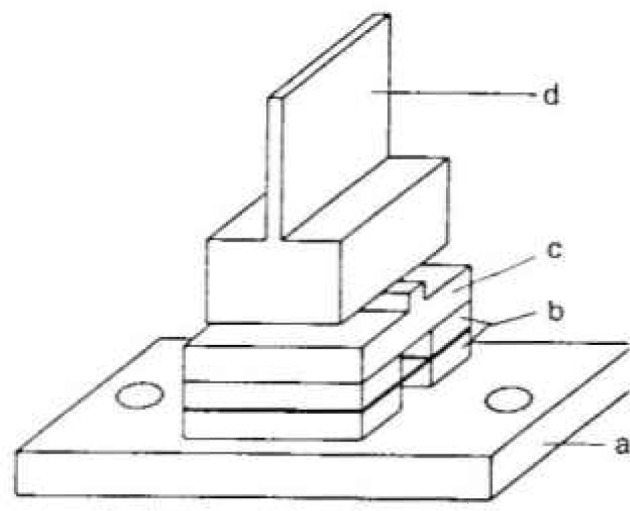
Detailed drawing of the second crystal, a – cast iron b – PZT, c – Si support, d – Si diffraction crystal.

In order to obtain strain free samples, the crystals are made with a 1.5 cm × 1.5 cm base on top of which is the thin 1.5 cm high wafer which is used for diffraction. Drawings of the long first crystal and the second crystal are shown in Figs. 5 and 6. Typical lengths of the long and short crystals are 6.3 cm and 1.2 cm, respectively. The long crystal is balanced on a 1.5 mm wide silicon rod which is centered on an optically polished silicon cylinder. This assembly is waxed together with a low temperature optician’s wax. The polished silicon cylinder is attached to a ground cast iron base with epoxy resin. The cast iron base and the rotating table are joined by screw fasteners. After assembly the angular offset between the normal to the polished silicon cylinder and the crystal planes is measured using an x-ray spectrometer and an autocollimator. The long crystal is mounted on a flexure hinge on the rotating table and is tipped an amount equal to the measured offset. In this way the crystal planes are made parallel to the axis of rotation with an estimated uncertainty of 2 seconds.

The short crystal assembly consists of a ground cast iron base, a piezoelectric tipper, a silicon anvil (with a small hole for a thermistor), and the crystal. The base, PZT elements, and silicon anvil are attached by conductive epoxy and the crystal is balanced on the raised portion of the silicon anvil (1.5 mm wide) and attached by low temperature optician’s wax. This assembly is mounted on a flexure hinge on the translation table. Using x rays diffracted by the first crystal above and below the plane of dispersion, the planes of the second crystal are made parallel to those of the first crystal by the coarse mechanical adjustment and the fine PZT tipper.

The crystals are cut with a diamond saw and then polished to the desired thickness using a chemically assisted mechanical polishing solution [20]. Typical polishing rates are 10 μm per h and 50 μm to 70 μm are removed from each side to insure that no saw damage remains. Crystal thickness was measured using a coordinate measuring machine [21]. The reproducibility of the thickness measuring procedure was 1 μm–2 μm. Polishing the long first crystal so that the two areas used for diffraction had equal thickness was the most difficult task. Because the edges of the crystals tend to be too thin, the areas used for diffraction were at least 2 mm from any crystal edge. After polishing all surfaces of the crystal except for the polished surfaces were etched in HF/HNO_3_ to relieve strain.

## 5. Temperature Measurement and Control

Because the lattice parameter measurements are relative measurements, we required only accurate relative temperature values and not accurate absolute values. Since the expansion coefficient for silicon is 2.56 × 10^−6^/K, relative temperature measurements accurate to 4 mK suffice for 1 × 10^−8^ lattice parameter measurements. Six ultra-stable calibrated thermistor probes are used to measure the temperature of the crystals: two thermistors on the base of the long first crystal (one on each side) and one thermistor on the silicon anvil to which each of the four silicon crystals is attached. Vacuum grease is used to insure good thermal contact between the silicon and the thermistors. The six thermistors and an ultra-stable calibrated standard resistor (2.5 kΩ) [22] are connected in series and powered with a constant current source (≈ 1 ×10^−5^ A). A precision digital voltmeter is used to read the voltage drop across each thermistor and the standard resistor. The temperatures are read every 4 s, so the temperature for a measurement time of 20 s is an average of five measurements.

Before and after a critical measurement, the six thermistors are placed in a constant temperature bath in order to measure offsets between the thermistors. The offsets are typically a few mK and are stable to less than 1 mK over times long compared to the few weeks needed for a lattice parameter measurement.

The temperature of the laboratory is constant to within ~0.1 K over several days. For the measurement reported here the spectrometer was isolated from direct air currents within the laboratory by a cloth curtain. The curtain has subsequently been replaced by an insulated chamber. The crystals are covered with an aluminum thermal shield and two 20 mm thick styrofoam thermal shields. The x-ray tubes, motors, and detectors are outside the shields. The temperature difference between the first and second crystals is typically 0.1 K and can be varied by changing the temperature of the water used to cool the x-ray rubes. The temperature differences between the second crystals is only a few mK. Variation of the temperature differences (the critical quantity for relative measurements) over a 24 h period is typically less than 20 mK.

## 6. Data Analysis

The angular offsets, *β*_s_ and *β*_u_, which are needed to calculate Δ*d/d* from Eq. (2) are the angular separation of profiles recorded along the path (−) and path (+). The profiles are measured by stepping the axis through *N* discrete angles (typically *N* = 110) which are recorded as angle interferometer fringe numbers. For each angle the number of x-ray photons in each detector is counted for a fixed time (typically 10 s to 20 s). The time per scan is 40 min to 80 min. The fringe numbers for each profile are corrected in a point by point manner for the temperature difference between the first and second crystals. From the Bragg equation the dependence of the diffraction angle, *θ*, on temperature, *T*, is easily seen to be

$$d\theta /dT=-\alpha_{0}\tan\theta ,$$

where *α*_0_ = linear coefficient of thermal expansion of the crystal. For silicon *α*_0_ = 2.56 × 10^−6^/K. Let *θ_−_* and *θ_+_* be the angular settings of the first crystal for the path (−) and path (+), respectively, the rotation angle reference be the *y* axis, and clockwise and counter clockwise rotations be positive and negative respectively. When the Bragg condition is simultaneously satisfied at Crystals 1 and 2 and both crystals are at a reference temperature, *T*_0_, (see Fig. 1a and Fig. 8).

$$\theta_{-}(T_{0})=\theta_{B2}(T_{0})-\theta_{B1}(T_{0})$$

and

$$\theta_{+}(T_{0})=-\theta_{B2}(T_{0})+\theta_{B1}(T_{0}).$$

When crystal 1 is at temperature, T_1_, and crystal 2 is at temperature, *T*_2_, then these two equations become

$$\begin{array}{c} \theta_{-}(T_{0})=[\theta_{B2}(T_{2})-\theta_{B1}(T_{1})]-\frac{d\theta }{dT}(T_{1}-T_{2})=\\ \theta_{-}(T_{1},T_{2})-\frac{d\theta }{dT}(T_{2}-T_{1}) \end{array}$$

and

$$\begin{array}{c} \theta_{+}(T_{0})=-\theta_{B2}(T_{2})+\theta_{B1}(T_{1})+\frac{d\theta }{dT}(T_{2}-T_{1})=\\ \theta_{+}(T_{1},T_{2})+\frac{d\theta }{dT}(T_{2}-T_{1}), \end{array}$$

where we have assumed the crystals are of the same material.

The angles *θ_−_* for the path (−) are corrected by *−*d*θ/*d*T(T_2_−T*_1_) while the angles *0*_+_ for the path (+) are corrected by +d*θ/*d*T* (*T*_2_*−T*_1_). Since the profiles are recorded in fringe numbers, these angular corrections need to be converted to fringes before adding. The angle measuring interferometer is positioned within a few minutes of the zero degree angle for all the measured offsets. Using the interferometer equation discussed above, the angle to fringe conversion factor of 0.0401 sec/fringe at zero degree angle is determined and used to convert the angle corrections due to temperature differences into fringes.

The angular offsets between the profiles on the temperature corrected fringe scale have been determined by two different methods. In the first method the profiles were fit with a Lorentzian function using a nonlinear least squares procedure in which the position, intensity, width, and background are adjusted. The data with its pronounced wiggles and central spike is not well represented by the smooth Lorentzian function. However, by taking the differences, *R_±_*, between the recorded profiles, *I*_±_, and the smooth fitting functions, *L_±_*, the wiggles of the two profiles are emphasized.

$$R_{\pm }(\theta_{i})=(I_{\pm }(\theta_{i})-L_{\pm }(\theta_{i}))$$

The correlation function, *C*(Δ*θ*), for the two sets of residuals is computed.

$$C(\Delta \theta )=\sum_{i=1}^{N}R_{+}(\theta_{i})+R_{-}(\theta_{i}+\Delta \theta ),$$

where the sum is over the *N* data points of the profile.

The value of Δ*θ* for which *C*(Δ*θ*) is a maximum is the angular separation (offset angle *β*) of the two profiles. The uncertainty in the measurement of the angular separation οf the profiles is typically ≈ 6 × 10^−9^ rad.

In the second method the dynamical diffraction function was fit to the recorded profiles. The crystal thicknesses were fixed at 0.455 mm and the structure factors were taken from Refs. [14 and 15]. The only adjustable parameters in the fitting procedure were the position, the intensity and the background. The angular separation between the profiles is obtained as the difference between the fitted position of the two profiles. The measured angular separations obtained with the two methods agree within the measurement uncertainty. Because the first method is computationally simpler and more easily adapted to a small computer, it was used to obtain all of the results presented below.

A single lattice comparison run usually consists of 16 to 20 data scans, preceded and followed by alignment scans. The profiles are scanned by repeating the following sequence: unknown crystal – cw rotation, unknown crystal – ccw rotation, standard crystal – cw rotation, and standard crystal – ccw rotation. After determining the angular separation for each scan, the angular separations vs time of day for each crystal are fit with a variable order polynomial (usually 3). The constant (in time) angular offset between these two curves is the angular offset between the unknown and standard crystals for one data set. The scatter in the offsets is typically <6 × 10^−9^ rad provided the crystals are well aligned and the same areas of the crystals are used to diffract the x-ray beam.

## 7. Comparison of Two Silicon Samples

Two Si samples were prepared as described in Sec. 4 from material acquired by PTB and NIST for absolute lattice parameter measurements. The NIST sample was supplied by Dow Chemical^2^ and was a slab adjacent to the NIST x-ray/optical interferometer. The PTB material was supplied by Wacker-Chemitronic and labeled WASO 17 by PTB. The relative difference between WASO 17 and WASO 4.2 (the PTB x-ray/optical interferometer crystal) has been measured to be 2.5 ± 1 × 10^−8^ [23](WASO 17–WASO 4.2). The first long crystal was prepared from material supplied by Monsanto.

These two samples were chosen because the lattice spacing of the NIST sample has been measured absolutely and the lattice spacing of WASO 17 was measured relative to the WASO 4.2 sample which was measured absolutely at the PTB. In the case of the NIST sample, further refinement of the absolute lattice parameter measurement is continuing [24, *25*]. Because the absolute lattice measurements have not yet achieved 0.01 ppm uncertainty, they do not provide as definitive a test of the Δ*d* measurements as is desirable. The published results of the absolute lattice spacing measurements are

$$d_{220}(\text{WASO}\;4.2)\;\text{at}\;22.5{}^{\circ}C=0.192015560\;(12)\;\text{nm}\;(\pm 6{.2\times 10}^{-8})$$

$$d_{220}(\text{NIST})\;\text{at}\;22.5{}^{\circ}C=0.19201554\;(2)\;\text{nm}\;(\pm 10{.4\times 10}^{-8})$$

which lead to

$$\Delta d/d(\text{NIST-WASO}\;4.2)=-1\times 10^{-7}\pm 1.2\times 10^{-7}.$$

In order to develop confidence in the Δ*d* measurements, we have measured the long term reproducibility by recording an extensive set of data over more than one half year during which the crystals were realigned on the spectrometer several times. In addition, measurements were taken using two different wavelengths (Ag Kα and Mo Kα x-ray radiation) and with the long first crystal in the two possible orientations (the 0° and the 180° orientation).

**Fig. 7:**
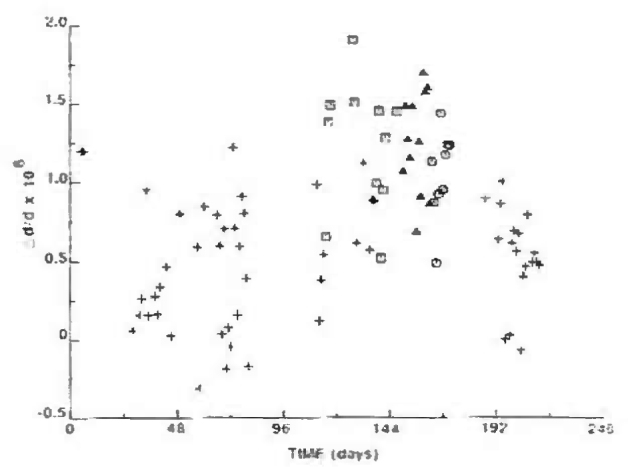
Comparison of NIST and WASO 17 crystals: + AgKα, first crystal 0°; □ AgK*α*, first crystal 180°; ⊙ MoKα, first crystal 0°; Δ MoK*α*, first cryslal 180°.

**Fig. 8:**
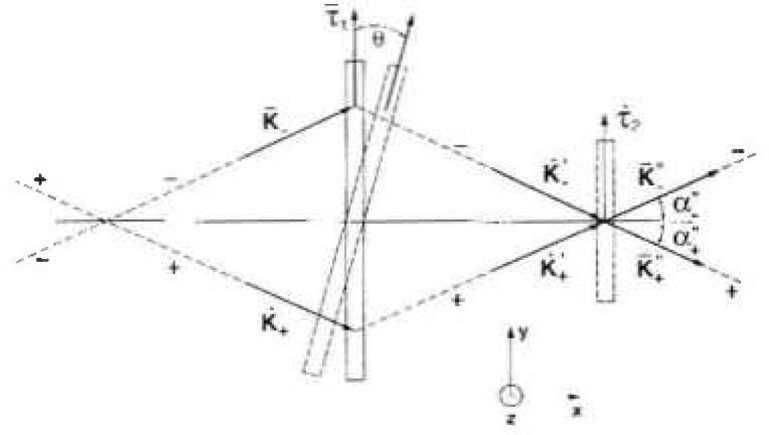
Geometry of the lattice parameter measurement with no crystal misalignment.

In Fig. 7 the measured values of Δ*d/d* are plotted vs time with different symbols for the different sources and different first crystal orientations. Note the long time span over which the Ag Kα, 0° orientation measurements were taken. In Table 1 the average numerical values for the different sources and first crystal orientations are presented. From both the table and the figure, the magnitude of systematic effects related to the source and first crystal orientation is estimated to be ~7 × l0^−9^. Table 2 provides a summary of uncertainty contributions resulting from systematic effects associated with 1) the wavelength and first crystal orientation, 2) crystal temperature measurements, 3) crystal misalignments, and 4) periodic nonlinearity in the Michelson angle interferometer.

The uncertainty in the measurement of the crystal temperature differences is less than 1 mK. This value was determined by periodically placing all of the thermistors in a constant temperature bath as explained in Sec. 5. A 1 mK uncertainty in the crystal temperature differences leads to an uncertainty of 3 × 10^−9^ in Δ*d/d*. The uncertainty introduced by crystal misalignment is discussed in detail in Appendix A and is estimated to be less than 2 × 10^−9^.

Heterodyne Michelson interferometers are prone to sub-periodic nonlinearity resulting from imperfect separation of the two frequencies by the primary beam splitter [26, 27]. The periodic nonlinearity can be evaluated by pressure scanning the interferometer and recording the fringe advance vs pressure increase. The maximum amplitude of the nonlinearity is estimated to be 4 × 10^−9^ rad. This means that the angular separation of two points on a diffraction profile which are separated by 0.5 interferometer fringe (4 × 10^−7^ rad) might be in error by 8 × 10^−9^ rad. However, because the profiles are typically 25 interferometer fringes wide, the influence of the interferometer periodic error on the profile peak position is significantly reduced by averaging. In addition, the phase relation of the x-ray profile to the angle interferometer fringes changes so that uncertainty due to periodic nonlinearity becomes part of the statistical uncertainty. We estimate that the uncertainty contribution to Δ*d/d* due to the systematic effect associated with the periodic nonlinearity of the angle interferometers is not larger than 5 × 10^−9^. The angle interferometer is being modified in order to reduce the periodic nonlinearity by approximately a factor of 5.

A final value for the NIST-WASO 17 comparison was obtained by considering each of the entries in Table 1 to be an independent measurement of equal weight. The statistical uncertainty was combined with the uncertainties from systematic effects in Table 2 to obtain a value for Δ*d/d* (NIST-WASO 17) = (1.037 ± 1.0) × 10^−8^. By combining this value with the WASO 17-WASO 4.2 difference noted above, one obtains

$$\Delta d/d(\text{NIST-WASO}\;4.2)=(3.5\pm 1.4)\times 10^{-8}.$$

This value is only slightly outside the 1 *σ* uncertainty of the absolute measurements. In addition, it should be remembered that the NIST absolute value is a preliminary result.

## 8. Conclusions

A lattice comparison facility has been established at NIST which is capable of measuring crystal lattice spacing difference with an uncertainty < 0.01 ppm. By using crystals of equal thickness, the recorded profiles exhibit fine structure which permits more precise measurement of the small angular offsets between profiles. The spectrometer is designed to permit easy interchange of crystal samples and the comparison of four samples in one setup. Procedures are provided for precise alignment of the crystals so that alignment errors contribute <2 × 10^−9^. A comparison of samples whose lattice spacings have been absolutely measured is consistent with the absolute measurements. In the near future other crystal samples (including Get destined for x- and gamma-ray diffraction will be compared using the spectrometer and the technique described here.

## Figures and Tables

**Fig. 9:**
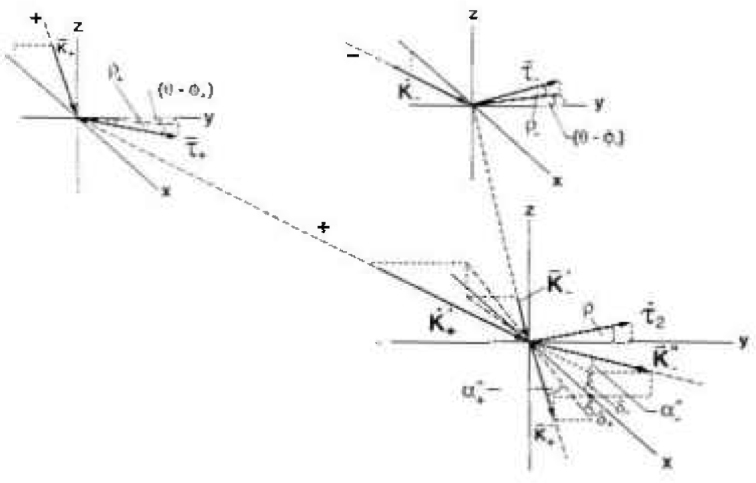
Geometry of the lattice parameter measurement including crystal misalignment.

**Fig. 10:**
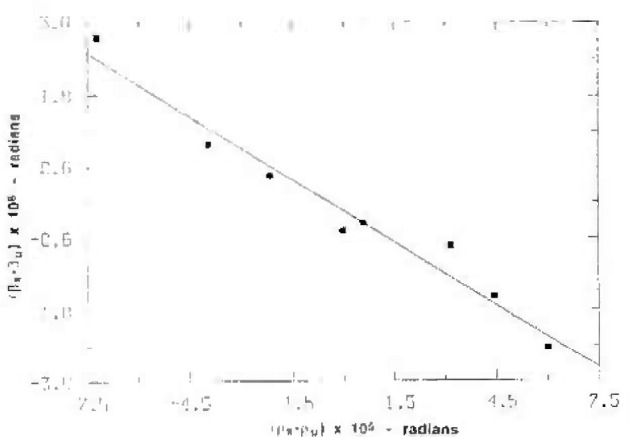
Measured data profile offsets, *β*_s_*−β*_u_, as a function of the measured crystal tilts (*ρ*_s_*−ρ*_u_). See text for more explanation.

**Fig. 11:**
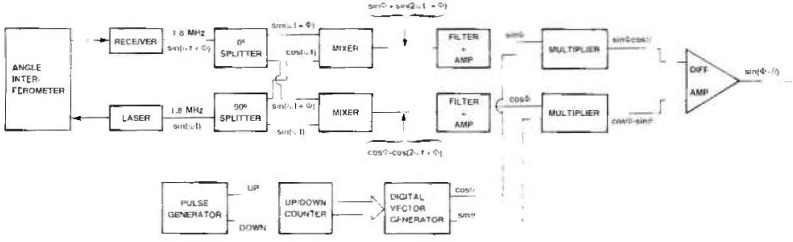
Block diagram of electronics used to measure whole and fractional fringes.

**Table 1:** Comparison of NIST and WASO 17 samples

Source	First crystal orientation	Δ*d/d ×* 10^8^ NIST-WASO 17
AgK*α*	0°	0.508 ± 0.379
AgK*α*	180°	1.236 ± 0.393
Mo K*α*	0°	1.050 ± 0.262
Mo K*α*	180°	1.251 ± 0.310

**Table 2:** Estimated uncertainties from systematic effects

Systematic effect	Contribution to Δ*d/d ×* 10^8^
λ and 1st crystal orientation	0.7
Temperature	0.3
Crystal misalignment	0.2
Interferometry nonlinearity	0.5

## Footnotes

2 Certain commercial equipment, instruments, or materials are identified in this paper to specify adequately the experimental procedure. Such identification does not imply recommendation or endorsement by the National Institute of Standards and Technology, nor does it imply that the materials or equipment identified are necessarily the best available for the purpose.

## 9. Appendix A, Derivation of the Equation for Calculation of Δ*d*

If the lattice planes in crystal 1 and 2 are a perfectly parallel with the axis of rotation and the x-ray beams lie in a plane normal to the axis corotation, then the difference in lattice spacing between crystal 1 and 2 can be derived from Bragg’s law (see Sec. 2). In reality, however, the alignment of the crystals and/or the x-ray beams is never perfect. In this appendix, equations for the calculation of Δ*d* which include misalignment terms are derived. The x-ray beams are described be wavevectors, ***k***, and the crystals are described be reciprocal vectors, *τ.* The derivation is based of the fact that the angular positions, *θ_+_* and *θ*_−_, of the symmetric rocking curve profiles, measured along path (+) and path (−) in the two crystal spectrometer, are equal to the two particular rotation angles *θ*, for which the Laue-conditions for path (+) and path (−), are simultaneously satisfied at crystal 1 and 2 (see Figs. 8 and 9).

Larson [28] has made a similar geometrical analysis of a lattice parameter comparison using Bragg geometry. We first derive the equation for Δ*d/d* for the ideal case involving perfect crystals and no misalignment of the crystals or the x-ray beams. The more experienced reader may choose to skip over this section. Next, we derive the equations for the nonideal case and apply them to “alignment scans” and “data scans.”

### 9.1 Ideal Case

First consider the ideal case of no misalignment of the x-ray beams or tilting of the crystals. The geometry of the measurement is then entirely confined to the *xy* plane and is as shown in Fig. 8. The coordinate system is such that the *z*-axis is parallel to the *θ*-axis (i.e., the axis of the rotating table), the *x*-axis is parallel to the lattice planes of interest in crystal 2, the *y*-axis is normal to the lattice planes in crystal 2, and the normal to the lattice planes in crystal 1 lies in the *xy* plane. The rotation angle reference is taken to be the *y*-axis and the *θ*’s, the angles of rotation of the first crystal, are positive for clockwise rotation and negative for counterclockwise rotation.

The x-ray beams are described by their wave vectors: ***k***_−_, ***k***_+_ for the incident rays; $k\prime_{-}$, $k\prime_{+}$ for the singly diffracted rays; and $k{\prime \prime }_{-}$, $k{\prime \prime }_{+}$ for the double diffracted rays. Using the angles shown in Fig. 8, the doubly diffracted rays can be written as

$$k{\prime \prime }_{-}=2\pi /\lambda (\cos\alpha {\prime \prime }_{-},\sin\alpha {\prime \prime }_{-},0),$$

$$k{\prime \prime }_{-}=2\pi /\lambda (\cos{\alpha^{\prime \prime }}_{+},\sin\alpha {\prime \prime }_{+},0).$$

The crystals are described by their reciprocal lattice vectors τ_1_(*θ*) for crystal 1 and *τ*_2_ for crystal 2 which can be written as

$$\tau_{1}(\theta )=\frac{2\pi }{d_{1}}(\sin\theta ,\cos\theta ,0),$$

$$\tau_{2}=\frac{2\pi }{d_{2}}(0,1,0).$$

The planes at the two ends of crystal 1 are assumed to be parallel and to have the same lattice spacing. When the Laue condition is satisfied at crystal 1

$$k\prime_{-}-k_{-}=-\tau_{1}(\theta_{-})\;\text{and}\;k\prime_{+}-k_{+}=\tau_{1}(\theta_{+}).$$ (3)

Similarly when the Laue condition is satisfied at crystal 2

$$k{\prime \prime }_{-}-k\prime_{-}=\tau_{2}\;\text{and}\;k{\prime \prime }_{+}-k\prime_{+}=-\tau_{2}.$$ (4)

Since the wave vectors have equal magnitude, 2*π*/*λ*, Eqs. (3 and 4) imply

$$\begin{array}{c} \tau_{1}(\theta_{-})\cdot (\tau_{2}-{k^{\prime \prime }}_{-})\frac{1}{2}{\left|\tau_{1}(\theta_{-})\right|}^{2}\text{and}\;\tau_{1}(\theta_{+})\cdot (\tau_{2}+k{\prime \prime }_{+})=\\ \frac{1}{2}{\left|\tau_{1}(\theta_{+})\right|}^{2}. \end{array}$$ (5)

By substituting the reciprocal lattice vectors and the wave vector withangle at the crystal 2 into Eq. (5) and using the Bragg equation, *λ* =2*d*_2_ sin*θ*_2_, to eliminate the wavelength, two equations for the lattice spacing difference are obtained.

$$\begin{array}{c} \frac{d_{2}-d_{1}}{d_{1}}=-\sin\theta_{-}\cot\theta_{B2}-(1-\cos\theta_{-})\approx \\ -\theta_{-}\cot\theta_{B2}-\frac{1}{2}\theta_{-}^{2} \end{array}$$

$$\begin{array}{c} \frac{d_{2}-d_{1}}{d_{1}}=-\sin\theta_{+}\cot\theta_{B2}-(1-\cos\theta_{+})\approx \\ \theta_{+}\cot\theta_{B2}-\frac{1}{2}\theta_{+}^{2} \end{array}$$

where terms up to second order in *θ_±_* have been retained. These two equations can be added to obtain

$$\frac{d_{2}-d_{1}}{d_{1}}=-\frac{\left(\theta_{-}-\theta_{+}\right)}{2}\cot\theta_{B2}-\frac{1}{8}{\left(\theta_{-}-\theta_{+}\right)}^{2},$$ (6)

where we have used the fact that *θ*_+_ *= −θ_−_.*

For comparisons in which the first and second crystal are made out of the same material [(*d*_2_−*d*_1_)/*d*_1_<10^−5^], the last term is negligible as the following inequality shows

$$\begin{array}{c} 10^{-10}>{\left|\frac{d_{2}-d_{1}}{d_{1}}\right|}^{2}>\frac{1}{2}{\left|\frac{d_{2}-d_{1}}{d_{1}}\right|}^{2}\tan^{2}\theta_{B2}\approx \\ \frac{1}{8}{\left(\theta_{-}-\theta_{+}\right)}^{2},\;\text{if}\;\theta_{B2}<54{}^{\circ}. \end{array}$$

If crystal 2 is alternately the standard and unknown crystal, the Eq. (6) becomes

$$\frac{d_{s}-d_{1}}{d_{1}}\approx -\frac{\beta_{s}}{2}\cot\theta_{\mathrm{Bs}\;}\text{where}\;\beta_{s}=\theta_{-s}-\theta_{+s}$$

$$\frac{d_{u}-d_{1}}{d_{1}}\approx -\frac{\beta_{u}}{2}\cot\theta_{\mathrm{Bu}\;}\text{where}\;\beta_{u}=\theta_{-u}-\theta_{+u}$$

These two equations immediately lead to Eq. (2).

$$\frac{d_{s}-d_{u}}{d_{1}}\approx -\frac{\beta_{s}-\beta_{u}}{2}\cot\theta_{\mathrm{Bs}}$$ (7)

### 9.2 Non-Ideal Case

Misalignment of the planes of crystals 1 and 2 with respect to the rotation axis, imperfections in the first crystal (i.e., lattice spacing gradient and nonparallelism of the planes at the two ends of the crystal), and the misalignment of the x-ray beams with respect to the plane normal to the rotation axis introduce correction terms to the measured lattice spacing difference. In the analysis which takes into account misalignments and crystal imperfections, the geometry of Fig. 9 is used.

The coordinate system is identical to that used for the ideal case, i.e., the *z* axis is parallel to the axis of rotation and the *x* axis parallel to the lattice planes in crystal 2. However, the wave and reciprocal lattice vectors no longer lie in the *xy* plane. The wave vector of the double diffracted x-rays can be put in the following form:

$$k{\prime \prime }_{\pm }=\frac{2\pi }{\lambda }(\cos\alpha_{\pm }\cos\delta_{\pm },\mp \sin\alpha_{\pm },\cos\alpha_{\pm }\sin\delta_{\pm }),$$ (8)

where *δ_±_* is the angle between the *xy* plane and the projection of the wavevector $k{\prime \prime }_{\pm }$ on the *xz* plane.

The reciprocal lattice vectors associated with path (+) and path (−) in crystal 1, *τ*_+_ and *τ*_−_, are no longer parallel and of equal magnitude. The two vectors *τ_±_* can be described by the six parameters *d*_±_, *ρ*_±_ and *θ_±_* as follows:

$$\begin{array}{l} \tau_{\pm }=\frac{2\pi }{d_{\pm }}(\sin(\theta -\phi_{\pm })\cos\rho_{\pm },\\ \cos(\theta -\phi_{\pm })\cos\rho_{\pm },\sin\rho_{\pm }), \end{array}$$ (9)

where *d_±_* is the lattice spacing in the diffracting region of crystal 1 corresponding to path (±), *ρ*_±_ is the angle between *τ*_±_ and the *xy*-plane, and *ϕ*_±_ is the particular angle of rotation *θ* for which *τ*_±_ is parallel to the *yz* plane. For a perfect first crystal, *d_+_* =*d*_−_, *ρ*_+_ *= ρ*_−_, and *ϕ_+_* = ϕ_−_. In crystal 2 the reciprocal lattice vector, *τ*_2_, is the same for both paths and is given by

$$\tau_{2}=\frac{2\pi }{d_{2}}(0,\cos\rho ,\sin\rho ),$$ (10)

where *d*_2_ is the lattice spacing in the diffracting region of crystal 2, and *ρ* is the angle between *τ*_2_ and *xy*-plane. Note that *τ*_2_ is in the *yz* plane.

For path (±), the relations between the wave vectors ***k***_±_ of the incident rays, $k\prime_{\pm }$ of the single diffracted rays, and $k{\prime \prime }_{\pm }$ of the double diffracted rays are given by the Laue conditions:

$$k\prime_{\pm }-k_{\pm }=\pm \tau_{\pm }\;(\text{crystal}\;1)$$ (11)

$$k{\prime \prime }_{\pm }-k\prime_{\pm }=\mp \tau_{2}\;(\text{crystal}\;2)$$ (12)

Since the wave vectors have equal magnitude, 2*π*/*λ*, Eqs. (11) and (12) imply that

$$\tau_{2}\cdot k{\prime \prime }_{\pm }=\mp \frac{1}{2}{\left|\tau_{2}\right|}^{2}$$ (13)

$$\tau_{\pm }\cdot (\tau_{2}\pm k{\prime \prime }_{\pm })=\frac{1}{2}{\left|\tau_{\pm }\right|}^{2}.$$ (14)

When the Laue condition is satisfied at crystal 2, the angles, *α_±_*, can be expressed as *α*_±_ = *θ*_B2_ + *ϵ*_±_ where *ϵ*_±_ are the small angular corrections to the second crystal Bragg angle because of crystal imperfections and misalignments. Thus Eq. (8) becomes

$$\begin{array}{c} k{\prime \prime }_{\pm }=\frac{2\pi }{\lambda }(\cos(\theta_{B2}+\epsilon_{\pm })\cos\delta_{\pm },\\ \pm \sin(\theta_{B2}+\epsilon_{\pm }),\cos(\theta_{B2}+\epsilon_{\pm })\sin\delta_{\pm }). \end{array}$$ (15)

In order to establish a relation between the lattice spacing *d* of crystal 2 and the rotation angles *θ* = *θ*_±_, for which the Laue-conditions corresponding to path (±) are simultaneously satisfied at crystal 1 and crystal 2, Eqs. (9), (10), and (15) are expanded to second order in the angles *θ*_±_−*ϕ*_±_, *ρ*_±_, *ρ* and *δ*_±_ and to first order in the correction angle *ϵ*_±_:

$$\tau_{\pm }=\frac{2\pi }{d_{\pm }}(\theta_{\pm }-\phi_{\pm },1-\frac{1}{2}{\left(\theta_{\pm }-\phi_{\pm }\right)}^{2}-\frac{1}{2}\rho_{\pm }^{2},\rho_{\pm })$$ (16)

$$\tau_{2}=\frac{2\pi }{d_{2}}(0,1-\frac{1}{2}\rho^{2},\rho )$$ (17)

$$k{\prime \prime }_{\pm }=\frac{\pi }{d_{2}}\left((1-\frac{1}{2}\delta_{\pm }^{2})\cot\theta_{B2}-\epsilon_{\pm },\;\mp 1\mp \epsilon_{\pm }\cot\theta_{B2},\delta_{\pm }\cot\theta_{B2}\right).$$ (18)

In the last equation, the wavelength *λ* has been eliminated using *λ* = 2*d*_2_sin*θ*_B2_. By insertion of Eqs. (17) and (18) into Eq. (13), an expression for the correction angles *ϵ*_±_ is easily obtained:

$$\epsilon_{\pm }=\frac{1}{2}\rho^{2}\tan\theta_{B}\pm \rho \delta_{\pm }.$$ (19)

Now by inserting Eqs. (16), (17), (18), and (19) into Eq. (14) the following equation can be derived:

$$\frac{d_{2}-d_{\pm }}{d_{\pm }}=\pm (\theta_{\pm }-\phi_{\pm })\cot\theta_{B}+e_{\pm },$$ (20)

where *e*_±_ is the second order correction term

$$\begin{array}{c} e_{\pm }=-\frac{1}{2}{\left(\theta_{\pm }-\phi_{\pm }\right)}^{2}-\frac{1}{2}(\rho_{\pm }-\rho )(\rho_{\pm }-3\rho )\\ \pm \delta_{\pm }(\rho_{\pm }-\rho )\cot\theta_{B2}. \end{array}$$ (21)

The angle *δ*_±_ in Eq, (21) describes the inclination of the double diffracted beam relative to the *xy*-plane, (see Fig. 9). If crystal 1 and crystal 2 are perfectly parallel, the wavevectors ***k****_±_*, $k\prime_{\pm }$, and $k{\prime \prime }_{\pm }$ are coplanar and the inclinations *δ*_±_ are determined simply by the positions of the source and detector slits. When the two crystals are not perfectly parallel, *δ_±_* are also dependent on the misalignment angles *ρ_±_* and *ρ.*

Let *ℓ*, *ℓ*′, and *ℓ″* be projections along the *x*-axis of the *x*-ray paths from the source slit to crystal 1, from crystal 1 to crystal 2, and from crystal 2 to the detector slit, respectively. The distances are shown in Fig. 1 and for our spectrometer are:

$$\begin{array}{l} \ell =(25.4\cos\theta_{B}+8.25)\;\mathrm{cm}\\ \ell^{\prime }=8.25\;\mathrm{cm}\\ \ell^{\prime \prime }=(28.5\;\cos\theta_{B})\;\mathrm{cm} \end{array}$$

If Δ*Z*_±_ denotes the difference in *Z*-coordinate between the detector slit and source slit of path (±), then the inclination *Δ*_±_ relative to the *xy*-plane of the straight line joining the two slits is given by:

$$\Delta_{\pm }=\frac{\Delta Z_{\pm }}{\ell +\ell^{\prime }+\ell^{\prime \prime }}.$$ (22)

The change in *Z*-coordinate as one moves from the source slit to the detector slit along path (*±*) can be expressed by combining the *Z*-components of the wave vectors ***k***_±_, $k\prime_{\pm }$, and $k{\prime \prime }_{\pm }$, normalized to unity and the distances traveled:

$$\begin{array}{c} \Delta Z_{\pm }=\frac{\lambda }{2\pi }\left[{\left(k_{\pm }\right)}_{z}\ell +{\left(k\prime_{\pm }\right)}_{z}\ell^{\prime }+{\left(k{\prime \prime }_{\pm }\right)}_{z}\ell^{\prime \prime }\right]\\ \frac{1}{\cos\theta_{B}}. \end{array}$$ (23)

From Eqs. (11), (12), and (15) and using Eqs, (9) and (10), the *Z*-components of the wave vectors are found to second order in *ρ*_±_, *ρ*, and *δ*_±_:

$$\frac{\lambda }{2\pi }{\left(k_{\pm }\right)}_{z}=\delta_{\pm }\cos\theta_{B}\pm 2(\rho -\rho_{\pm })\sin\theta_{B}$$ (24)

$$\frac{\lambda }{2\pi }{\left(k\prime_{\pm }\right)}_{z}=\delta_{\pm }\cos\theta_{B}\pm 2\rho \sin\theta_{B}$$ (25)

$$\frac{\lambda }{2\pi }{\left(k{\prime \prime }_{\pm }\right)}_{z}=\delta_{\pm }\cos\theta_{B}.$$ (26)

By insertion of Eqs. (22), (24), (25), and (26) into Eq. (23) a relation between *δ_±_* and Δ_±_ is obtained:

$$\delta_{\pm }=\Delta_{\pm }\pm 2\left(\frac{\ell }{\ell +\ell^{\prime }+\ell^{\prime \prime }}\rho_{\pm }-\frac{\ell +\ell^{\prime }}{\ell +\ell^{\prime }+\ell^{\prime \prime }}\rho \right)\tan\theta_{B}.$$

Using this expression, the unknown angles *δ_±_* can be eliminated from Eq. (21):

$$\begin{array}{c} e_{\pm }=-\frac{1}{2}{\left(\theta_{\pm }-\phi_{\pm }\right)}^{2}+2(\rho_{\pm }-\rho )(a_{1}\rho_{\pm }+b_{1}\rho )\pm \\ \Delta_{\pm }(\rho_{\pm }-\rho )\cot\theta_{B} \end{array}$$ (27)

where

$$a_{1}=\frac{\ell }{\ell +\ell^{\prime }+\ell^{\prime \prime }}-\frac{1}{4}=0.23$$

$$b_{1}=\frac{\ell^{\prime \prime }}{\ell +\ell^{\prime }+\ell^{\prime \prime }}-\frac{1}{4}=0.15.$$

Even though the distances *ℓ* and *ℓ*″ depend on the Bragg angle *θ*_B_, the numerical values for *a*_1_ and *b*_1_ are essentially identical for the two x-ray sources (*θ*_B_=16.9° for Ag K*α*_1_ and *θ*_B_ = 21.6° for Mo K*α*_1_).

When crystal 1 and 2 are both of the same material, an upper limit of the first term in Eq. (27) is easily estimated:

$$\begin{array}{c} \frac{1}{2}{\left(\theta_{\pm }-\phi_{\pm }\right)}^{2}<{\left|\frac{d-d_{\pm }}{d_{\pm }}\right|}^{2}<10^{-10}\\ 10^{-10}>{\left|\frac{d-d_{\pm }}{d_{\pm }}\right|}^{2}>\frac{1}{2}{\left|\frac{d-d_{\pm }}{d_{\pm }}\right|}^{2}\tan^{2}\theta_{B}\approx \frac{1}{2}{\left(\theta_{\pm }-\phi_{\pm }\right)}^{2},\\ \text{if}\;\theta_{B}<54{}^{\circ}. \end{array}$$

This term is two orders of magnitude smaller than the intended uncertainty (a few parts in 10^8^) in the lattice spacing comparison and is therefore neglected in the following. The remaining two terms in Eq. (27) are both zero, if the lattice planes in crystal 1 and 2 are parallel, i.e., *ρ*_+_−*ρ* = 0 and *ρ*_−_−*ρ* = 0. The aim of the alignment scan is to measure *ρ_+_*−*ρ* and *ρ*_−_−*ρ* and then to adjust the tip of crystal 2 so as to make *ρ_+_*−*ρ* and *ρ*_−_−*ρ* as close to zero as possible.

#### 9.2.1 The Alignment Scan

In the alignment scan, only one path, path (+) or path (−), is used, Two rocking curves are measured simultaneously: one curve using the upper (up) position and one using the tower (lo) position of the detector slit. For the path (−), the observed difference Δ*θ*_−_ in the centers of the two rocking curves (Δ*θ*_−_ = *θ*_−up_−*θ*_−lo_), the change in the inclination (*Δ*_−up_−*Δ*_−lo_) when the detector slit is moved from the upper to the lower position, and the difference in misalignment of the two crystals, *ρ*_−_−*ρ*, are related. The following equation is derived directly from Eqs. (20) and (27):

$$\rho_{-}-\rho =\frac{-\Delta \theta_{-}}{\Delta_{-\text{up}}-\Delta_{-\mathrm{lo}}}.$$ (28)

Similarly, for path (+) it follows that

$$\rho_{+}-\rho =\frac{-\Delta \theta_{+}}{\Delta_{+\text{up}}-\Delta_{+\mathrm{lo}}}$$ (29)

(*Δ*_−up_*−Δ*_−lo_) and (*Δ*_+up_*−Δ*_+lo_) can be estimated from mechanical measurements of the spectrometer and are ~9 × 10^−3^ rad. Due to bending of crystal 1, *ρ*_+_ and *ρ_−_* are not equal, Consequently, the sample crystal can only be aligned for one of the two paths. If the tilt of crystal 2 is adjusted so that |*Δθ*_−_| ≈ 2 × l0^−8^ rad, then typical values for |*Δθ_+_*| are 1 × 10^−7^ rad. These values imply that

$$\begin{array}{l} \left|\rho_{-}-\rho \right|\approx 2\times 10^{-6}\\ \left|\rho_{+}-\rho \right|\approx 1\times 10^{-5}. \end{array}$$

The magnitude of the individual *ρ*’s can also be controlled and estimated. By rotating the 1st crystal by 180°, the tilt of the first crystal can be adjusted so that *ρ*_+_ and *ρ*_−_ are < 1 × 10^−5^ rad.

#### 9.2.2 The Data-Taking Scan

In the data-taking scan, two rocking curves are simultaneously measured with the detector slits fixed in the middle position: one curve using path (+), and one using path (−). We define the average lattice spacing, *d*_1_, in crystal 1 as

$$d_{1}=\frac{2d_{+}d_{-}}{d_{+}+d_{-}},$$

and average Eqs. (20) and (27) over path (+) and path (−) to obtain

$$\frac{d_{2}-d_{1}}{d_{1}}=\frac{1}{2}(\theta_{+}-\theta_{-}+\eta_{0})\cot\theta_{B}+e,$$ (30)

where

$$\begin{array}{c} e=\{(\rho_{+}-\rho )(a_{1}\rho_{+}+b_{1}\rho )+(\rho_{-}-\rho )(a_{1}\rho_{-}+b_{1}\rho )\}+\\ \frac{1}{2}\{\Delta_{+}(\rho_{+}-\rho )-\Delta_{-}(\rho_{-}-\rho )\}\cot\theta_{B} \end{array}$$ (31)

and

$$\eta_{0}=\phi_{-}-\phi_{+}.$$ (32)

The angle *η*_0_ describes a bending of crystal 1 around the *θ*-axis and is independent of the particular sample used as crystal 2. Obviously, the appearance of the unknown angle *η*_0_ in Eq. (30) excludes the possibility of using crystal 1 as an absolute reference crystal.

The two crystals being compared are placed on the translation table and are used alternately as crystal 2. One of the crystals serves as the standard (s) and one as the unknown (u). The difference in lattice spacing, Δ*d = d*_s_*−d*_u_ for the two sample crystals can be derived from Eqs. (30) and (31).

$$\frac{\Delta d}{d_{1}}=-\left(\frac{\beta_{s}-\beta_{u}}{2}\right)\cot\theta_{B}+\Delta e$$ (33)

where:

$$\begin{array}{l} \beta_{s}=\theta_{-s}-\theta_{+s}\\ \beta_{u}=\theta_{-u}-\theta_{+u} \end{array}$$

$$\begin{array}{l} \Delta e=[a_{2}(\rho_{+}+\rho_{-})+b_{2}(\rho_{s}+\rho_{u})-\frac{1}{2}\\ (\Delta_{+}-\Delta_{-})\cot\theta_{B}](\rho_{s}-\rho_{u})=\\ \left[a_{2}(\rho_{+}+\rho_{-})+2b_{2}\rho_{u}-\frac{1}{2}(\Delta_{+}-\Delta_{-})\cot\theta_{B}\right] \end{array}$$

$$(\rho_{s}-\rho_{u})+b_{2}{\left(\rho_{s}-\rho_{u}\right)}^{2},$$ (34)

where *a_2_=b*_1_−*a*_1_= −0.08 and *b_2_*=−*2b*_1_= −0.30.

In order to make Δ*e* small, the tilts of the crystals are adjusted so that *ρ*’s are < 1 × 10^−5^ (see Sec. 9.2.1). Care is taken to center the source and detector slits on the plane of dispersion in order to make *Δ_+_−Δ_−_* small, but mechanical measurements suggest that the magnitude of the *Δ*’s is ⩽ 3 × 10^−4^. The correction term can be estimated by measuring (*β*_s_*−β*_u_) as a function of (*ρ*_s_*−ρ*_u_) where *ρ*_u_ is kept fixed and *ρ*_s_ is varied. For *ρ*_s_*−ρ*_u_ ⩽ 5 × l0^−5^, Eqs. (33) and (34) can be approximated by

$$\frac{\Delta d}{d_{1}}=-\left(\frac{\beta_{s}-\beta_{u}}{2}\right)\cot\theta_{B}+C(\rho_{s}-\rho_{u})$$

where

$$C=a_{2}(\rho_{+}+\rho_{-})+2b_{2}\rho_{u}-\frac{1}{2}(\Delta_{+}-\Delta_{-})\cot\theta_{B}.$$

A plot of (*β*_s_*−β*_u_) vs (*ρ*_s_*−ρ*_u_) is shown in Fig. 10 and has an approximate slope of *−*3.6 × 10*^−^*^4^ in reasonable agreement with the estimates of the *Δ*’s from the mechanical measurements and the estimates of the individual *ρ*’s.

Before and after data scans are recorded using a standard and unknown crystal, alignment scans are recorded for the standard and unknown crystal also. The quantity *ρ*_s_*−ρ*_u_ is determined from the alignment scans and is maintained at a value < 4 × 10*^−^*^6^ rad. Thus the correction term Δ*e* < 2 × 10*^−^*^9^. Correction of the data for measured values of Δ*e* does not reduce the statistical spread of repeated measurements. We have thus chosen to increase the relative uncertainty of the final results by 2 × 10*^−^*^9^, but not to correct individual measurements. Although the correction term is small, it could be reduced by an order of magnitude by making the source and detector slits adjustable about the plane of dispersion. Modifications to the spectrometer to permit this adjustment are in progress.

## 10. Appendix B. Electronics Associated with the Interferometer

The layout of the Michelson angle interferometers is shown in Fig. 2 and is briefly described in Sec. 3. In this appendix the electronics used to measure the whole and fractional fringes and to servo-control the rotation axes are described. A block diagram of the electronics is provided in Fig. 11.

The 1.8 MHz TTL level signals from the laser and the detector are first converted to low impedance signals by buffer amplifiers. The laser beat signal is then passed through a 2-way 90° splitter with 0° and 90° split yielding sin (*ωt*) and cos (*ωt*), respectively. The detector beat signal is passed through a 2-way 0° splitter yielding two sin (*ωt + ϕ*) signals. The laser’s cos (*ωt*) and one of the detector’s sin (*ωt + ϕ*) are combined in a mixer which gives signals at the sum and difference frequencies. The difference frequency signal is sin ϕ and the sum frequency signal is sin (2*ωt* + *ϕ*) which is attenuated by a low pass filter. Meanwhile the sin (*ωt*) from the laser is combined with the other sin (*ωt* + *ϕ*) of the detector in another mixer which after filtering gives cos ϕ. The sin ϕ and cos ϕ signals are next amplified to restore their signal strengths which have deteriorated due to losses in the splitters, mixers, and filters.

Ninety degree changes in ϕ which correspond to ~0.04 second rotation of the rotary table are detected by sending the sin ϕ and cos ϕ signals to an up/down counter. Smaller changes in ϕ are measured by comparing ϕ to a standard angular signal (sin *θ* and cos *θ*) obtained as the output of a digital vector generator. A vector generator with a few 0.1° sensitivity provides a total angular sensitivity of a few × 10^−4^ seconds with the vector generator address being directly related to the fringe fraction. By taking the difference of the product of sin ϕ and cos *θ* and the product of cos *ϕ* and sin *θ* the signal sin(*ϕ−θ*) is obtained. This is an appropriate error signal for locking the angle of the rotary table via a piezoelectric transducer to the position where *ϕ = θ.*
