# Erratum: Precision Comparison of the Lattice Parameters of Silicon Monocrystals

**DOI:** 10.6028/jres.099.025

**Published:** 1994

**Authors:** E. G. Kessler, A. Henins, R. D. Deslattes, L. Nielsen, M. Arif

**Affiliations:** National Institute of Standards and Technology, Gaithersburg, MD 20899-0001

[J. Res. Natl. Inst. Stand. Technol. Volume 99, Number 1, January–February 1994, p. 1]

The figures over figure captions 2, 3, and 4 have been permuted. The figure over figure caption 4 belongs with figure caption 2. The figure over figure caption 2 belongs with figure caption 3. The figure over figure caption 3 belongs with figure caption 4.

